# Clinical Significance of Endometrial Cells in Pap Smear of Women Aged 40 Years and Older

**DOI:** 10.5146/tjpath.2022.01570

**Published:** 2022-09-15

**Authors:** Esra Keleş, Uğur Kemal Öztürk, Cihat Murat Alınca, Serkan Akış, Canan Kabaca, Handan Çetiner

**Affiliations:** Department of Gynecologic Oncology, Zeynep Kamil Training and Research Hospital, Istanbul, Turkey; Department of Pathology, University of Health Sciences, Zeynep Kamil Training and Research Hospital, Istanbul, Turkey

**Keywords:** Endometrial cells, Endometrial cancer, Papanicolaou (Pap) test, The Bethesda System

## Abstract

***Objective:*** To investigate the histopathological follow-up results in women diagnosed with endometrial cells in the Papanicolaou (Pap) test.

***Material and Method:*** Between January 2013 to December 2018, women with endometrial cells on the Pap test were searched from the hospital electronic database. The patients with endometrial cells on the Pap test who underwent further histopathological evaluation and who were followed-up for at least 1 year were enrolled in the study, while those who had a Pap test result other than endometrial cells, were lost during follow-up, or had missing data were excluded.

***Results:*** Out of 91,142 Pap smears, 121 (0.1%) cytologically had endometrial cells, and of those 65 cases were eligible for final analysis. The mean age of patients with premalignant/malignant lesions (57.7 ± 2.9) was higher than those with benign lesions (50.1 ± 0.7), with 77% of them in the postmenopausal period. Gynecologic premalignant/malignant lesions were detected in 9 (17.7%) patients including 2 (3.1%) endometrial hyperplasias and 7 (10.8%) endometrial cancers. The menopausal status (p=0.010) and being 50 years and older (p=0.002) were significantly associated with pre-neoplastic or neoplastic changes in patients with endometrial cells.

***Conclusion:*** The presence of endometrial cells in Pap tests may be a harbinger of endometrial pathologies, especially at the age of 50 years and over. The menopausal status is another possible determinant in detecting endometrial carcinoma. Further investigation may be suggested in women aged ≥50 years and postmenopausal in the event of endometrial cell detection.

## INTRODUCTION

Endometrial cancer is the most common female genital tract malignancy in developing countries (1). Although the Papanicolaou (Pap) test has been a valid test for screening cervical carcinoma and its precursor lesions for many years, there is currently no non-invasive methods to screen for endometrial cancer.

Whether the presence of endometrial cells (ECs) in cervicovaginal smear has any clinical value has been investigated by researchers for years. The Bethesda System (TBS) 1991 proposed notifying the asset of spontaneously exfoliated, benign-appearing endometrial cells only in postmenopausal women as an epithelial cell abnormality (2). The TBS 2001 suggested age-based reporting without the need for clinical evaluation and determined the age limit of reporting ECs as over 40 years (3). The TBS 2014 updated its approach for the age limit of reporting ECs from 40 to 45 years (4). With the application of these guidelines on ECs and the transition of conventional cytology to liquid-based cytology in cervical screening testing techniques, ECs have been more frequently noticed, assisting in the detection of endometrial cancers (5,6).

Since several studies have addressed endometrial cells in the early diagnosis of endometrial carcinomas, we aimed to investigate the relationship of initial ECs cytology results with histopathological results and patient demographic characteristics.

## MATERIAL and METHOD

We retrospectively searched our institutional electronic database for the records of all women aged 40 years and older who had ECs on the Pap test between January 1, 2013, and December 31, 2018. This study was approved by the Research Ethics Committee and was conducted in accordance with the ethical standards described in an appropriate version of the 1975 Helsinki Declaration, revised in 2000 (Approval number: 2019/18).

Demographic and histopathologic follow-up findings, including pipelle aspiration biopsies, endocervical curettages and/or hysterectomies within twelve months after the index Pap smear were collected from the hospital electronic medical records. The ThinPrep (Hologic Inc., Marlborough, Mass., USA) assay was used for liquid-based cytologic examination.

Women aged 40 years and older, with cytological findings compatible with endometrial cells, and who had at least one-year of follow-up were included in the study. Women who were lost during follow-up or had missing data, and those who had a Papanicolaou test result other than endometrial cells were excluded from the study.

Since our institution is specifically focused on obstetrics and gynecology, our pathologists are experienced in gynecopathology. All pathological specimens were performed by specialized gynecopathologists. The standard was set based on ‘The Bethesda System For Reporting Cervical Cytology’. According to the Bethesda System, benign endometrial cells were evaluated in Pap smears among women over 40 years of age. A diagnosis of benign ECs was made based on the findings of three-dimensional clusters with small and round nuclei, indistinct nucleoli, and scant cytoplasm with indistinct cell borders which was identified with the ‘The Bethesda System For Reporting Cervical Cytology’.

The patients were analyzed with respect to age (40-49, ≥50 years). The histopathological follow-up findings were classified as benign, pre-malignant, and malignant. Endometrial atrophy, endometrial breakdown, proliferative endometrium, hormonal effect, endometritis, leiomyoma, adenomyosis, and endometrial polyp were defined as benign pathologies. Simple endometrial hyperplasia with atypia, simple endometrial hyperplasia without atypia, complex endometrial hyperplasia with atypia, and complex endometrial hyperplasia without atypia were defined as pre-malignant pathologies. Any malignant neoplasm was defined as a malignant pathology.

All statistical analyses were performed using the Statistical Package for the Social Science (IBM SPSS version 25) for Windows software. Percentage and frequency values for categorical variables, and mean (± standard deviation) for quantitative variables were used in descriptive statistics of the data. The relationship between categorical variables was performed using Fisher’s exact test and Chi-Squared test, where appropriate. The independent sample t-test was used to compare quantitative data. In this study, the type I error rate was set at 0.05. A p-value ≤ 0.05 was considered statistically significant.

## RESULTS

**Figure 1:**
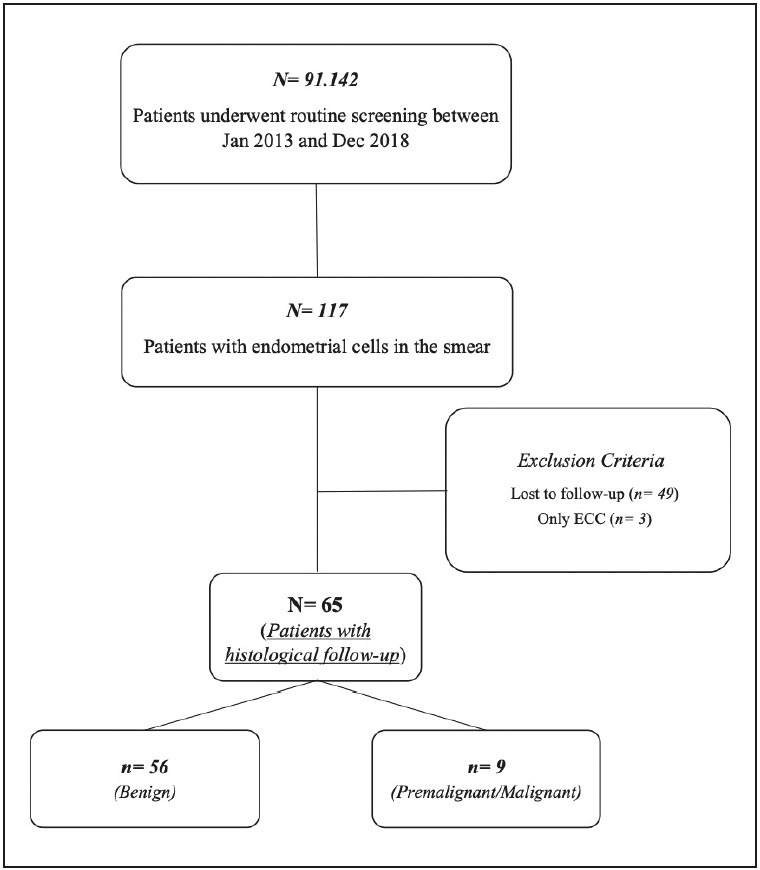
The flow diagram of the study.

A total of 91,142 Pap smears were screened in the study period. Of these, 117 (0.1%) contained endometrial cells. After excluding the patients who did not meet the eligibility criteria, 65 patients with ECs remained for the final analysis. The flow diagram of the study is shown in Figure 1.

Fifty-six patients were assigned to the benign group and nine patients were assigned to the premalignant/malignant group. There was a significant difference between histopathological findings and age groups (p=0.002). The mean age of patients with premalignant/malignant lesions (57.7±2.9) was significantly higher than those with benign lesions (50.1±0.7) (p=0.033). Seven (77.8%) cases in the premalignant/malignant group were in the postmenopausal period, while 39 cases (69.6%) in the benign group were in the reproductive age (p=0.010). The menopausal status was significantly associated with pre-neoplastic or neoplastic changes in postmenopausal endometrial tissue. The patients’ detailed demographic and clinical characteristics according to the pathology results are shown in Table 1.

**Table 1:** Analysis of factors according to pathology results.

		**Pathology Results**		
	**N/%**	**Benign** **(n=56) (86.1%) (n/%)**	**Premalignant/Malignant** **(n=9) (13.9%) (n/%)**	**p**
Age Groups				
40-49	32 (49.2)	32 (57.1)	0 (0.0)	**0.002**
≤50	33 (51.8)	24 (42.9)	9 (100.0)	
Age (years)*	51.2 ± 0.8	50.1 ± 0.7	57.7 ± 2.9	**0.033a**
Reproductive Function				
Reproductive Age	41 (63.1)	39 (69.6)	2 (22.2)	**0.010**
Postmenopausal Age	24 (36.9)	17 (30.4)	7 (77.8)	

Statistical analyses were performed with Fisher’s Exact Test. a= Performed by Independent Samples T-Test. *= Mean and S.E. (Standart Error) values are used.

**Figure 2:**
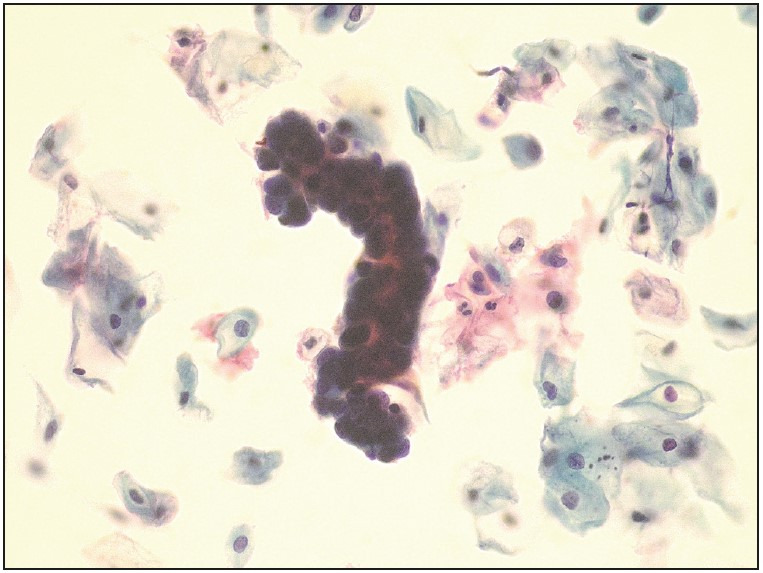
Cluster of endometrial cells reported as benign in ThinPrep smear preparation (Papanicolaou, x400).

**Figure 3:**
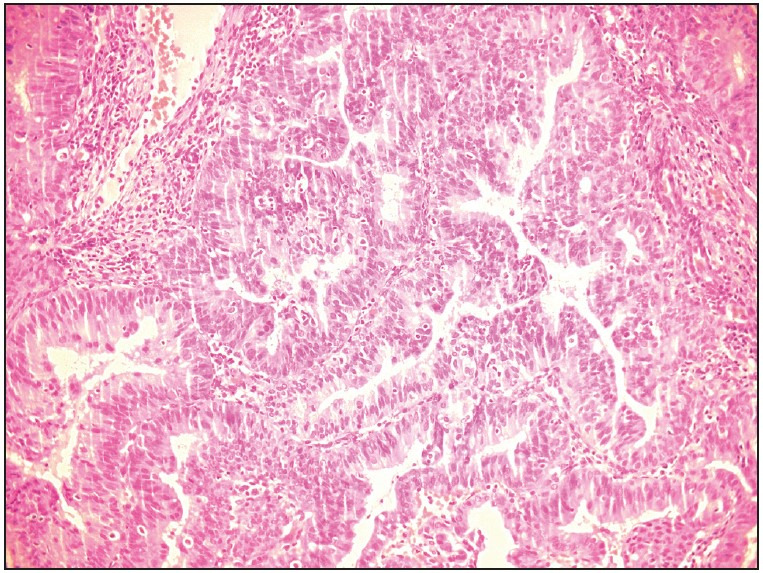
Histology of the patient’s ‘well differentiated endometrial adenocarcinoma’ (Hematoxylin-Eosin, x400).

Histopathological findings were evaluated between the age groups of 40-49 (n=32) and ≥50 (n=33) years. There was a significant difference with respect to endometrial cancer in women aged 50 and over (p=0.024) and endometrial polyps in women aged 40-49 (p=0.047) years. Pre-malignant and malignant lesions diagnosed in a total of 9 cases were as follows: 2 patients were diagnosed with endometrial hyperplasia, 2 patients were diagnosed with endometroid endometrial carcinoma (Figure 2 Figure 3), and 5 patients were diagnosed with non-endometroid endometrial carcinoma. Histopathological results of women with endometrial cells by age group are shown in Table 2.

**Table 2:** Histopathological results of women with benign endometrial cells by age groups.

	**Age Groups**		
	**40-49** **(n/%)**	**≥50** **(n/%)**	**p**
Benign (Unspecified)	9 (28.1)	12 (36.4)	0.478
Proliferative Endometrium	10 (31.3)	6 (18.2)	0.221
Endometrial Polyps	13 (40.6)	6 (18.2)	**0.047**
Endometrial Hyperplasia	0 (0.0)	2 (6)	0.492*
Endometrial Cancer	0 (0.0)	6 (18.2)	0.024*
Uterine Sarcoma	0 (0.0)	1 (3)	1.000*

Statistical analyses were performed with the Chi-Squared Test. *= Performed with Fisher’s Exact Test.

## DISCUSSION

In the present study, we examined the relationship between the endometrial cells in Pap smears with the patient’s histopathological findings and demographic characteristics. The study revealed that menopausal status and being aged ≥50 years were possible factors that may be useful in detecting endometrial pathologies. The study also implied the need for further comprehensive investigation in women aged 50 years and over and who were postmenopausal in the event of endometrial cell detection.

The Global Cancer Observatory 2020 data has revealed 417367 new corpus uteri cancer cases worldwide, leading to 97370 deaths annually (7). The majority of endometrial cancers can be diagnosed early due to abnormal uterine bleeding symptoms; however, very few are asymptomatic. Therefore, the presence of endometrial cells in Pap tests can be helpful in discovering neoplastic endometrial tissue changes in this group.

The reporting age of endometrial cells is still a controversial issue. There was no significant difference in premalignant/malignant lesions between women aged 45 years and over and those aged 40-44, in which endometrial cells were detected in the Pap test. Therefore the reporting age of ECs was increased from 40 to 45 in the TBS 2014 guideline (4). Some studies have argued that the evaluation of the endometrial cells in the Pap test according to the menopausal state or the presence of symptoms is more valuable in detecting pathologies (8,9). Thereupon, studies have been proposed involving different age limits and various variables (8,10–15). In our study, no pathological lesions were found in women between the ages of 40-45 and 46-49, in contrast to women aged 50 and over, most of whom were postmenopausal and had pre-neoplastic and neoplastic lesions. In all, these results suggest that further investigation may be beneficial in women aged 50 and over and postmenopausal in the event of endometrial cell detection.

Endometrial cells in the Pap test have received much attention in the detection of endometrial pathologies. The significance of demographic and clinical characteristics in the prediction of malignancy in women with benign endometrial cells has not yet been dealt with in depth. Therefore, our paper calls into question whether there are any other relevant factors besides ECs reported in cervical cytology that could help in detecting significant pathologies. In this context, we tried to examine the age and menopausal status of the patients to increase the chance of discovering pre-neoplastic and neoplastic lesions. In our view, our results constitute a step towards enhancing the knowledge on the importance of ECs’ association with malignancy. Given that our findings are based on a limited number of women with ECs, these results thus need to be interpreted with care and validated with larger sample-sized studies.

While the reporting age of endometrial cells has been updated over the years, potential risks such as patients’ anxiety, health care costs, excessive surgical procedures, and complications that may occur in these interventions have been carefully weighed besides the benefits of early diagnosis (16). Increasing the reporting age limit for ECs from 45 to 50 increases the likelihood of detecting malignant tumors, but on the other hand, fails to discover some early stages of malignant lesions (15). Our study findings that ECs in Pap tests were significant in detecting endometrial pathologies in patients 50 years and older are consistent with previous studies (9–11,14). Additional large-scale multicenter studies are needed to support our findings in the future.

We are aware that our research may have two limitations. The first is that it is a single-center, small sample-sized study. Unfortunately, we were unable to obtain relevant data on the patients’ symptoms, the medications, the use of intrauterine devices, and the day of the menstrual period on which uterine sampling was performed due to the retrospective nature of the study. The strengths of the study were that it constituted the experience of one of the largest tertiary institutions in the field of obstetrics and gynecology in the country, and experienced gynecologic pathologists evaluated the histopathological specimens. However, we believe that our results will provide a comprehensive flow of information into the growing body of literature on the importance of reporting endometrial cells.

In conclusion, endometrial cells in Pap test could be a useful aid for clinicians in the early diagnosis of endometrial pathologies. Further evaluation with additional diagnostic workup may therefore be beneficial in postmenopausal women aged 50 years and older.

## Conflict of Interest

The authors have no conflicts of interest to declare.

## Funding

None.
